# Association Between Preoperative Steroid Use and Short‐Term Outcomes Following Pancreaticoduodenectomy: A Nationwide Analysis Using the National Clinical Database

**DOI:** 10.1002/ags3.70286

**Published:** 2026-10-19

**Authors:** Yoshito Tomimaru, Hideki Endo, Hiroyuki Yamamoto, Teruhide Ishigame, Hideki Ueno, Ken Shirabe, Hidetoshi Eguchi

**Affiliations:** ^1^ Department of Gastroenterological Surgery, Graduate School of Medicine The University of Osaka Suita Japan; ^2^ Department of Healthcare Quality Assessment, Graduate School of Medicine The University of Tokyo Tokyo Japan; ^3^ Department of Health Data Science Tohoku University Graduate School of Medicine Sendai Miyagi Japan; ^4^ The Japanese Society of Gastroenterological Surgery Tokyo Japan; ^5^ Department of Hepato‐Biliary‐Pancreatic and Transplant Surgery and Fukushima Medical University Fukushima, Fukushima Japan; ^6^ Department of Surgery National Defense Medical College Tokorozawa Saitama Japan; ^7^ Division of Hepatobiliary and Pancreatic Surgery, Department of General Surgical Science, Graduate School of Medicine Gunma University Maebashi Gunma Japan

**Keywords:** chronic steroid use, nationwide database, pancreatic fistula, pancreaticoduodenectomy

## Abstract

**Background:**

Pancreaticoduodenectomy (PD) carries a high risk of postoperative complications. Although steroids are widely prescribed for various chronic diseases, the specific impact after PD remains unclear. This study evaluated the association between preoperative chronic steroid use and short‐term outcomes following PD.

**Methods:**

This retrospective cohort study used Japanese National Clinical Database data from 2019 to 2022. Patients undergoing PD were categorized by preoperative chronic steroid use. To account for varying background factors, three multivariable regression models with different adjustment levels (Models 1–3) were constructed.

**Results:**

Of 40 878 patients, 521 (1.3%) had chronic steroid use prior to PD. The steroid group had higher crude rates of adverse outcomes, including postoperative pancreatic fistula (POPF; 30.7% vs. 22.1%) and severe complications (7.7% vs. 4.4%). In the most extensively adjusted multivariable model (Model 3), preoperative steroid use remained a significant factor associated with POPF (odds ratio [OR] 1.80, 95% confidence interval [CI] 1.47–2.20) and severe complications (OR 1.71, 95% CI: 1.22–2.40). The adverse impacts on infectious complications and surgery‐related mortality were also consistently significant across all models, whereas preoperative steroid use was not significantly associated with re‐operation in any of the models.

**Conclusions:**

Preoperative chronic steroid use is significantly associated with poor short‐term outcomes after PD, including POPF, severe complications, infectious complications, and mortality. These risks remained consistent regardless of adjustment level, highlighting the need for intensive perioperative management.

## Abstract 2

This nationwide retrospective cohort study using data from the Japanese National Clinical Database evaluated the association between preoperative chronic steroid use and short‐term outcomes in 40 878 patients undergoing pancreaticoduodenectomy. Multivariable regression analysis demonstrated that preoperative steroid use was significantly associated with increased risks of postoperative pancreatic fistula and major complications. These findings highlight that preoperative steroid use itself is a potent risk factor for poor short‐term outcomes, necessitating intensive perioperative risk management.

## 1 Introduction

Pancreaticoduodenectomy (PD) is one of the most technically challenging procedures in gastrointestinal surgery and is associated with a high incidence of postoperative complications. Common complications include postoperative pancreatic fistula (POPF), intra‐abdominal abscess, delayed gastric emptying, and postoperative hemorrhage, which can lead to prolonged hospitalization or even mortality [1, 2]. The occurrence of these complications can be influenced by the patient's overall health status and perioperative management, as well as the condition of the pancreas itself and the technical aspects of the surgery.

Systemic steroid therapy is commonly prescribed for a variety of chronic conditions, including autoimmune diseases, inflammatory disorders, allergic diseases, and immunosuppression after organ transplantation [3, 4]. Although steroids play an essential role in the management of these diseases, their use can lead to other conditions, such as central obesity, adrenal insufficiency, electrolyte imbalance, osteoporosis, and diabetes, contributing to patient frailty, which in turn results in an increased risk of postoperative complications in steroid‐treated patients. As a result, preoperative chronic steroid use has been recognized as a risk factor for postoperative complications after some types of surgery. Several studies have demonstrated an association between preoperative steroid use and unfavorable outcomes in various types of gastrointestinal surgery [5, 6, 7, 8, 9, 10, 11, 12]. However, the evidence regarding pancreatic resection remains limited [13, 14]. For example, in our previous single‐center study, chronic steroid use was found to be significantly associated with an increased risk of POPF after pancreatectomy [14]. However, the number of steroid‐treated patients in the study was small, implying that the evidence level remains insufficient to establish a generalized conclusion. Furthermore, previous studies could mostly report only crude risk associations due to limited sample sizes, leaving the true risk of steroids unclear without adequate adjustment for numerous perioperative confounding factors. Therefore, large‐scale analyses, such as those using national clinical databases, are necessary to provide robust evidence. The Japanese National Clinical Database (NCD) is a comprehensive, nationwide registry that includes detailed perioperative data from a wide range of surgical procedures. The NCD offers sufficient statistical power to assess risk factors and outcomes in various gastrointestinal surgeries. Thus, this study aimed to evaluate the association between preoperative steroid use and short‐term postoperative outcomes after PD using data from the NCD, with step‐by‐step adjustment for various clinical variables to minimize the influence of patient background.

## 2 Patients and Methods

### 2.1 Study Design and Data Source

This study is a retrospective cohort analysis based on data from the NCD. The NCD was established in 2011 in collaboration with the board certification system for surgery and has since expanded into a nationwide database covering more than 95% of surgeries performed across Japan. More than 5500 medical institutions participate, and more than 14 million cases have been registered in the past decade [15, 16, 17, 18]. The study was approved by the Institutional Review Board at The University of Osaka Hospital (Approval No. 25243).

### 2.2 Patients and Eligibility Criteria

This study included patients who underwent PD and were registered in the NCD between 2019 and 2022. This specific study period was selected to ensure the consistency of extracted variables, as registration formats and data specifications remained fully standardized during these years. Open, laparoscopic, or robot‐assisted PD were all eligible surgeries. Patients were excluded if they underwent emergency surgery, combined resection with other organs, or other concomitant surgical procedures, such as total gastrectomy, colectomy, hepatectomy, or adrenalectomy. However, patients who underwent portal vein or superior mesenteric vein (PV/SMV) resection and reconstruction were included in the study cohort. Patients with missing data in any of the evaluated variables were also excluded from the analysis, adhering to a complete case analysis approach. Missing data were minimal (*n* = 14, < 0.1%) because the NCD system mandates complete entry for core items, and laboratory variables were selectively restricted to parameters routinely measured in standard practice.

### 2.3 Variables

The primary exposure was preoperative chronic steroid use, defined in the NCD as the regular oral or intravenous administration of a steroid medication, such as prednisolone or dexamethasone, within 30 days prior to surgery for the treatment of chronic diseases. Topical application of steroids to the skin and the use of inhaled or rectal steroids were excluded from this definition. Patients who had received only a short‐term course (≤ 10 days) of steroids within 30 days before surgery were not included. The patients were divided into two groups according to the presence or absence of preoperative chronic steroid use: the steroid group and the non‐steroid group.

To evaluate patient backgrounds and potential confounders, we collected preoperative data, including age, sex, body mass index (BMI), comorbidities, laboratory data, and American Society of Anesthesiologists Physical Status (ASA‐PS) [19, 20]. Age was categorized using a threshold of 75 years in accordance with the standard definition of late‐stage elderly in Japan, and BMI was categorized using a threshold of 25 kg/m^2^ based on standard definitions for overweight. Comorbidities were defined according to NCD definitions: the presence of diabetes mellitus, dyspnea, impaired activities of daily living (ADL) requiring assistance within 30 days prior to surgery, encephalopathy within 30 days, ascites within 30 days, esophageal varices within 6 months, hypertension within 30 days, congestive heart failure within 30 days, angina pectoris within 30 days, history of cardiac surgery, symptomatic peripheral vascular disease (PVD), preoperative hemodialysis within 14 days, history of cerebrovascular disease, and body weight loss (defined as ≥ 10% loss in the past 6 months). Laboratory data included white blood cell count, hemoglobin, platelet count, albumin, total bilirubin, and creatinine, with thresholds defined based on standard institutional reference ranges for normal values. Surgical factors were also collected, including the surgical approach (open, laparoscopic, or robot‐assisted), operative time, intraoperative blood loss, and the requirement for intraoperative transfusion. Thresholds for prolonged operative time and large intraoperative blood loss were selected based on previously published pancreatic surgery literatures [21, 22, 23, 24].

The outcomes of this study were the incidence of POPF, defined as Grade B or greater according to the 2016 criteria set by the International Study Group of Pancreatic Fistula, and the incidence of severe postoperative complications, defined as Grade IIIb or greater according to the Clavien‐Dindo classification [25, 26]. This threshold of Grade IIIb or greater was specifically chosen to focus on critical morbidity requiring intervention under general anesthesia or intensive organ support. The study also evaluated the incidence of infectious postoperative complications, re‐operation, and surgery‐related mortality. Infectious postoperative complications were evaluated as a composite outcome comprising genuine infectious events, including superficial surgical site infection (SSI) at the skin incision, deep SSI at the fascial or muscle layer, organ or body cavity SSI, pneumonia, urinary tract infection, and postoperative sepsis. Notably, POPF and biliary leakage were not included in this endpoint, as these leakage events are not necessarily accompanied by active infection. Surgery‐related mortality was defined as death within 30 days postoperatively, regardless of hospitalization status, or in‐hospital death up to 90 days after surgery.

### 2.4 Statistical Analysis

Patient characteristics and outcomes were compared between the steroid and non‐steroid groups. As described above, missing values were handled using a complete case analysis, excluding 14 patients (0.03% of the total screened cohort) with missing variable data. All data were presented as numbers and percentages. Under the guidance of expert NCD biostatisticians, candidate covariates for multivariable models were preselected based on clinical relevance. Variable selection was restricted strictly to potential confounders. To evaluate the association between preoperative steroid use and postoperative outcomes, we employed mixed‐effects logistic regression models with a random intercept for each medical institution to account for clustering of patients within hospitals. We prespecified three nested models with sequential adjustment for covariate blocks as follows:

- Model 1 (base model), adjusted for age, sex, and facility‐level effects.
- Model 2, adjusted for Model 1 variables plus BMI and the following comorbidities: diabetes mellitus, dyspnea, impaired ADL requiring assistance within 30 days prior to surgery, encephalopathy within 30 days, ascites within 30 days, esophageal varices within 6 months, hypertension within 30 days, congestive heart failure within 30 days, angina pectoris within 30 days, history of cardiac surgery, symptomatic PVD, preoperative hemodialysis within 14 days, history of cerebrovascular disease, and body weight loss.
- Model 3, adjusted for Model 2 variables plus preoperative laboratory data: white blood cell count, hemoglobin, platelet count, albumin, total bilirubin, creatinine, and ASA‐PS.

This sequential modeling strategy was implemented to evaluate the robustness of the association between chronic steroid use and short‐term postoperative outcomes across different levels of covariate adjustment. Rather than relying on a single final model, we evaluated the trend of the risk estimates across these three distinct levels of adjustment to determine how the addition of different patient factors influenced the results. Variables with no events or extremely sparse counts in the chronic steroid group were not included as individual covariates in the multivariable models to avoid unstable estimation; specifically, encephalopathy was excluded because there were no events in the chronic steroid group (Table 1). All statistical analyses were carried out in R version 4.5.1 (2025; R Foundation for Statistical Computing, Vienna, Austria).

**TABLE 1:** Baseline characteristics and surgical outcomes of patients with and without preoperative chronic steroid use.

Factors	Total (*n* = 40 878)	Non‐steroid group (*n* = 40 357)	Steroid group (*n* = 521)
Preoperative factors			
Age ≥ 75 years	15 808 (38.7)	15 584 (38.6)	224 (43.0)
Sex, male	24 658 (60.3)	24 404 (60.5)	254 (48.8)
BMI ≥ 25 kg/m^2^	7601 (18.6)	7492 (18.6)	109 (20.9)
Comorbidities			
Diabetes mellitus	13 013 (31.8)	12 804 (31.7)	209 (40.1)
Dyspnea	309 (0.8)	284 (0.7)	25 (4.8)
Impaired ADL	774 (1.9)	747 (1.9)	27 (5.2)
Encephalopathy	20 (0.0)	20 (0.0)	0 (0.0)
Ascites	416 (1.0)	410 (1.0)	6 (1.2)
Esophageal varices	88 (0.2)	87 (0.2)	1 (0.2)
Hypertension	18 399 (45.0)	18 157 (45.0)	242 (46.4)
Congestive heart failure	111 (0.3)	108 (0.3)	3 (0.6)
Angina pectoris	381 (0.9)	374 (0.9)	7 (1.3)
History of cardiac surgery	469 (1.1)	459 (1.1)	10 (1.9)
Symptomatic PVD	83 (0.2)	82 (0.2)	1 (0.2)
Preoperative hemodialysis	226 (0.6)	218 (0.5)	8 (1.5)
History of cerebrovascular disease	1788 (4.4)	1767 (4.4)	21 (4.0)
Body weight loss	1770 (4.3)	1741 (4.3)	29 (5.6)
Laboratory data			
White blood cell ≥ 10 000/μL	1311 (3.2)	1246 (3.1)	65 (12.5)
Hemoglobin < 10.0 g/dL	3265 (8.0)	3206 (7.9)	59 (11.3)
Platelets < 15.0 × 10^4^/μL	3191 (7.8)	3145 (7.8)	46 (8.8)
Albumin < 3.5 g/dL	9737 (23.8)	9586 (23.8)	151 (29.0)
Total bilirubin ≥ 3.0 mg/dL	2720 (6.7)	2700 (6.7)	20 (3.8)
Creatinine ≥ 2.0 mg/dL	502 (1.2)	483 (1.2)	19 (3.6)
ASA‐PS ≥ 3	6174 (15.1)	6026 (14.9)	148 (28.4)
Surgical factors			
Approach			
Open	38 685 (94.6)	38 199 (94.7)	486 (93.3)
Laparoscopic	1446 (3.5)	1424 (3.5)	22 (4.2)
Robotic	747 (1.8)	734 (1.8)	13 (2.5)
Operation time > 500 min	14 043 (34.4)	13 867 (34.4)	176 (33.8)
Intraoperative blood loss > 500 mL	21 383 (52.3)	21 118 (52.3)	265 (50.9)
Intraoperative transfusion	8262 (20.2)	8128 (20.1)	134 (25.7)
Outcomes			
POPF	9083 (22.2)	8923 (22.1)	160 (30.7)
Severe complications	1800 (4.4)	1760 (4.4)	40 (7.7)
Infectious postoperative complications	7360 (18.0)	7212 (17.9)	148 (28.4)
Re‐operation	1486 (3.6)	1463 (3.6)	23 (4.4)
Surgery‐related mortality	644 (1.6)	620 (1.5)	24 (4.6)

*Note:* Values are *n* (%) unless otherwise indicated.

Abbreviations: ADL, activities of daily living; ASA‐PS, American Society of Anesthesiologists Physical Status; BMI, body mass index; POPF, postoperative pancreatic fistula; PVD, peripheral vascular disease.

## 3 Results

### 3.1 Patient Selection and Baseline Characteristics

The patient selection process is illustrated in Figure 1. A total of 47 155 patients who underwent PD between 2019 and 2022 were initially identified from the NCD. After excluding 293 patients who underwent emergency surgery, 5970 patients who underwent combined resection with other organs, and 14 patients with insufficient data in the evaluated variables, a final analytical cohort of 40 878 patients was established. Among these eligible patients, 521 (1.3%) were categorized into the steroid group and the remaining 40 357 (98.7%) into the non‐steroid group.

**FIGURE 1:**
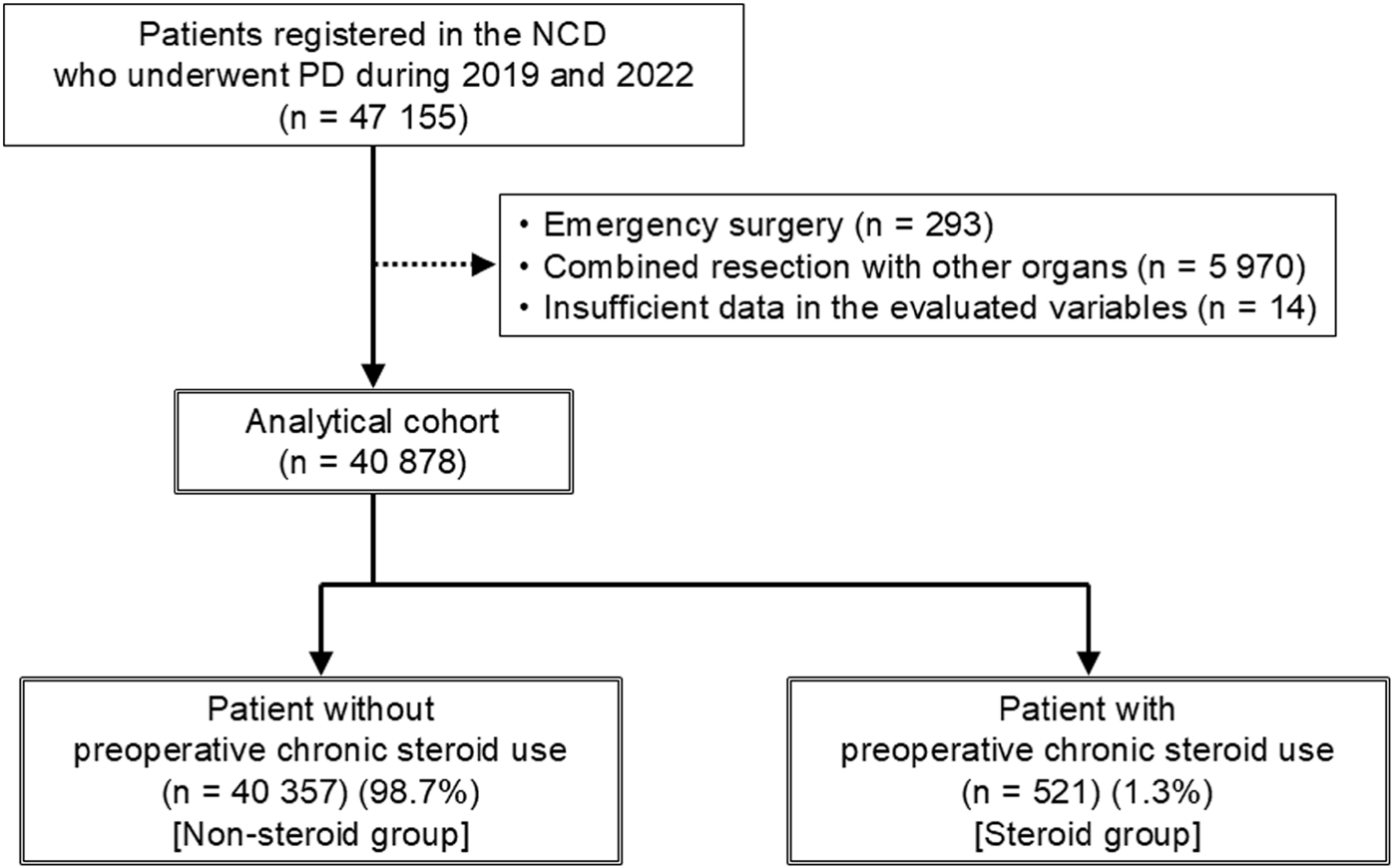
Flow chart of patient selection. This flowchart illustrates the process of selecting patients from the Japanese National Clinical Database (NCD). After applying the eligibility criteria, 40 878 patients undergoing pancreaticoduodenectomy (PD) were analyzed (steroid group: *N* = 521; non‐steroid group: *N* = 40 357).

The baseline demographics, including preoperative comorbidities and laboratory data, are summarised in Table 1. The steroid group had a higher proportion of elderly individuals (age ≥ 75 years) and a lower proportion of males (48.8% vs. 60.5%) compared with the non‐steroid group. Regarding comorbidities, the steroid group had a substantially higher prevalence of diabetes mellitus, dyspnea, and impaired ADL. Preoperative laboratory evaluations showed higher rates of elevated white blood cell count (≥ 10 000/μL), decreased hemoglobin (< 10.0 g/dL), decreased albumin (< 3.5 g/dL), and elevated creatinine (≥ 2.0 mg/dL). The rate of ASA‐PS grade ≥ 3 was also notably higher in the steroid group than in the non‐steroid group.

Surgical factors for both groups are also presented in Table 1. The distribution of the surgical approach was comparable between the two groups; the open approach was used in the vast majority of cases, followed by laparoscopic and robotic approaches. The proportions of patients experiencing a prolonged operative time (> 500 min) and large intraoperative blood loss (> 500 mL) did not differ between the groups. However, patients in the steroid group more frequently required intraoperative blood transfusions than those in the non‐steroid group.

### 3.2 Short‐Term Postoperative Outcomes in the Steroid and Non‐Steroid Groups

In the baseline comparison (Table 1), the steroid group experienced higher crude incidence rates for all evaluated adverse short‐term outcomes than the non‐steroid group. The steroid group had both a higher incidence of POPF and a higher rate of severe complications and infectious postoperative complications compared with the non‐steroid group. The re‐operation rate was also higher in the steroid group than in the non‐steroid group. Notably, a more than three‐fold increase in surgery‐related mortality was observed in the steroid group compared with the non‐steroid group (Table 1).

### 3.3 Mixed‐Effects Logistic Regression Analysis

Multivariable mixed‐effects logistic regression analyses revealed consistent associations between preoperative chronic steroid use and adverse short‐term outcomes across the three models (Figure 2). In the baseline model adjusted for age, sex, and institutional random effects (Model 1), preoperative steroid use was significantly associated with an increased risk of POPF (odds ratio [OR] 1.71, 95% confidence interval [CI] 1.40–2.08). This significant association remained stable and consistent even after adjusting for baseline comorbidities in Model 2 (OR 1.77, 95% CI: 1.44–2.16) and preoperative laboratory data and physical status in Model 3 (OR 1.80, 95% CI: 1.47–2.20). The significant association with severe complications was also sustained across all models. The risk estimate showed a minor decrease from Model 1 (OR 1.96, 95% CI: 1.41–2.73) through Model 2 (OR 1.79, 95% CI: 1.28–2.51) but remained significant in Model 3 (OR 1.71, 95% CI: 1.22–2.40). In addition, preoperative steroid use was a strong and remarkably stable factor associated with infectious complications regardless of the adjustment level, demonstrating consistent risk estimates from Model 1 (OR 1.87, 95% CI: 1.53–2.29) through Model 3 (OR 1.79, 95% CI: 1.45–2.20). Although the point estimates for the ORs were slightly elevated in the steroid group, the association with re‐operation did not reach significance in any of the models (Model 1: OR 1.27, 95% CI: 0.83–1.95; Model 2: OR 1.19, 95% CI: 0.77–1.84; Model 3: OR 1.16, 95% CI: 0.75–1.79). Preoperative chronic steroid use exhibited the strongest association with surgery‐related mortality. The baseline model (Model 1) showed a high risk (OR 3.61, 95% CI: 2.33–5.59), but the risk estimates gradually decreased as chronic comorbidities were controlled in Model 2 (OR 3.14, 95% CI: 2.00–4.92). In Model 3, which additionally controlled for preoperative laboratory variables and physical status, the association remained markedly high and significant (OR 2.86, 95% CI: 1.82–4.49). Collectively, the adverse effect of preoperative chronic steroid use on short‐term outcomes, except re‐operation, remained significant and robust regardless of the variations in covariate adjustment blocks.

**FIGURE 2:**
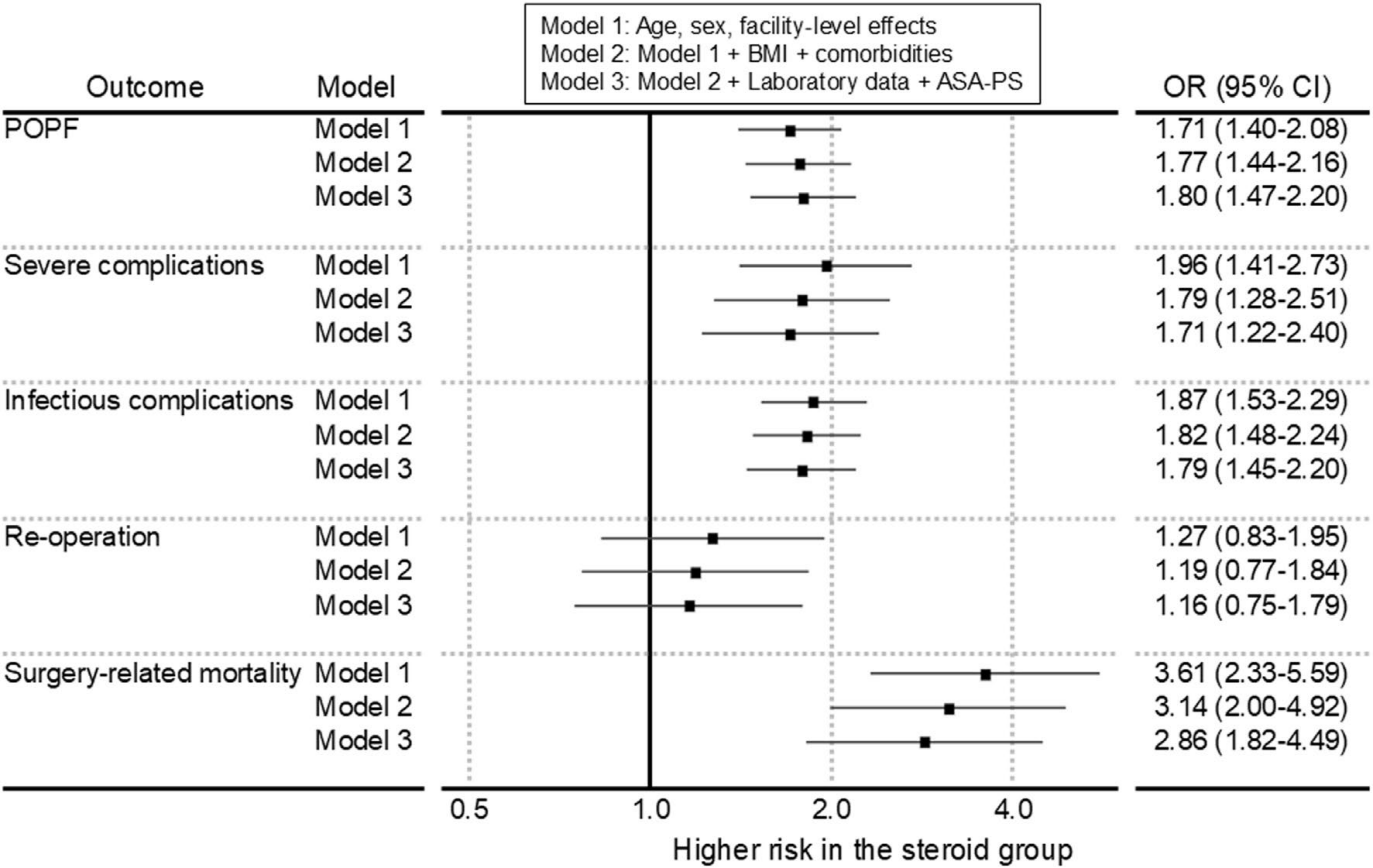
Forest plot of odds ratios (ORs) for postoperative outcomes across Models 1–3. ORs and 95% confidence intervals (CIs) are shown for three sequential multivariable mixed‐effects models. The vertical dashed line indicates an OR of 1.0. Each model incorporates step‐by‐step adjustment blocks as indicated in the figure. ASA‐PS, American Society of Anesthesiologists Physical Status; BMI, body mass index; POPF, postoperative pancreatic fistula.

## 4 Discussion

In this study, we evaluated the association between preoperative chronic steroid use and short‐term postoperative outcomes following PD using a large‐scale nationwide database in Japan. Our multi‐model regression analysis demonstrated that preoperative steroid use is robustly and consistently associated with several adverse outcomes, including POPF, severe complications, and infectious complications. Notably, the association was remarkably strong for surgery‐related mortality, which remained significant even after sequentially adjusting for baseline demographics, preoperative comorbidities, laboratory findings, and physical status. In contrast, no significant association was observed with the rate of reoperation.

The adverse effects of steroids on surgical outcomes have been reported for various gastrointestinal surgeries, including colorectal and gastric resections, primarily showing an increased risk of anastomotic leakage and wound‐related infectious complications [6, 8, 9, 10]. However, evidence specifically focusing on pancreatic resections has been extremely limited due to the low prevalence of chronic steroid users undergoing such high‐complexity procedures. Although our previous single‐center study suggested a potential link between chronic steroid use and an increased risk of POPF, the small sample size prevented a definitive, generalized conclusion [14]. Furthermore, because of the limited number of cases, almost all previous clinical studies could provide only crude risk assessments without the statistical capability to rule out the heavy influence of underlying conditions and baseline patient demographics. Against this background, the clinical significance and key novelty of the current study lie in its ability to clarify the multivariable‐adjusted association of steroids with outcomes by overcoming these sample size limitations using the NCD. Patients requiring chronic steroid therapy naturally carry a substantial burden of comorbidities, as shown by the higher rates of diabetes mellitus, impaired ADL, and poor preoperative laboratory data in our steroid group. By applying a step‐by‐step adjustment strategy rather than relying on a single final model, we tracked how the risk estimates shifted as different sets of patient variables were sequentially controlled. Importantly, the adjusted ORs for POPF, infectious complications, and surgery‐related mortality remained consistently elevated and significant across all three models. This stability across different levels of covariate adjustment highlights the robustness of our findings, indicating that preoperative steroid use itself is significantly associated with poor short‐term outcomes, playing a severe, distinct role in worsening short‐term outcomes rather than reflecting the patients' baseline clinical frailty. In contrast, a NSQIP study by Aziz et al. [13] evaluated preoperative steroid use in PD but found no significant associations with outcomes. Although definitions of steroid use (within 30 days) and eligibility were comparable, our study evaluated a substantially larger cohort (*n* = 40 878 vs. 5976), providing higher statistical power to demonstrate independent risk associations across multivariable models. Interestingly, the prevalence of steroid use was markedly lower in the NCD (1.3%) than in the NSQIP (10.4%), suggesting that Japanese patients requiring preoperative steroids may represent a distinct, highly vulnerable subgroup. Steroids are known to impair collagen synthesis, suppress early inflammatory responses necessary for tissue repair, and compromise systemic immune functions, which likely explains the persistently high rates of postoperative adverse events observed in this study [3, 4, 7].

From the standpoint of clinical application, these findings suggest a need for intensive perioperative care and risk stratification for chronic steroid users undergoing PD. Surgeons should recognize that these patients are at an exceptionally high risk of severe postoperative morbidity and mortality. For these patients, careful perioperative management is required. Moreover, when obtaining informed consent for the operation, clinicians can utilize the objective risk estimates provided by this large‐scale analysis to give patients a clearer understanding of the true perioperative risks. Recently, Mizuma et al. [27] published an NCD‐based study identifying risk factors, including long‐term steroid use, for POPF after PD using NCD data from 2019 to 2021 (*n* = 35 365). Because our study analyzed NCD data from 2019 to 2022 (*n* = 40 878), there is a substantial overlap in the study populations between the two studies. However, the research objectives and analytical methodologies differ fundamentally. While Mizuma et al. primarily focused on constructing an overall statistical prediction model incorporating broad database variables, our study was specifically designed to perform an evaluation focused on the clinical association of preoperative chronic steroid therapy across multiple short‐term outcomes, including POPF, severe complications, infectious complications, and surgery‐related mortality. Furthermore, developed under the direct guidance of expert NCD biostatisticians, our modeling strategy strictly focused on preselected clinical confounders while deliberately identifying potential intermediate variables on the causal pathway. This approach minimizes mediation bias and provides a refined, clinically robust assessment of the independent risk associated with chronic steroid therapy.

This study has several limitations. First, despite the comprehensive nature of the NCD, certain granular clinical data, such as the specific primary disease requiring steroid therapy, the exact daily dosage, cumulative dose, duration of therapy, and concomitant immunosuppressive medications, were not available. Consequently, our findings are restricted to the NCD‐defined variable of “preoperative chronic steroid use,” which may encompass a heterogeneous patient population. Second, specific risk factors related to the pancreas itself, including pancreatic texture and main pancreatic duct diameter, which are known to be critical predictors of POPF, are not routinely recorded in the database. Third, the retrospective nature of the study introduces inherent limitations regarding data collection, though the high coverage rate of the NCD minimizes selection bias.

In conclusion, this nationwide study demonstrated that preoperative chronic steroid use is consistently associated with poor short‐term outcomes following PD, particularly POPF, severe complications, infectious postoperative complications, and surgery‐related mortality. The elevated risks remained robust across different levels of adjustment for patient background. Surgical teams must approach these vulnerable patients with strict risk awareness and meticulous perioperative management strategies to mitigate these substantial risks.

## Author Contributions

**Yoshito Tomimaru:** conceptualization, methodology, writing – original draft. **Hideki Endo:** methodology, writing – review and editing, formal analysis. **Hiroyuki Yamamoto:** methodology, writing – review and editing, formal analysis. **Teruhide Ishigame:** methodology, formal analysis, writing – review and editing. **Hideki Ueno:** resources, supervision, writing – review and editing. **Ken Shirabe:** writing – review and editing, supervision, resources. **Hidetoshi Eguchi:** conceptualization, methodology, supervision, writing – review and editing.

## Funding

The authors have nothing to report.

## Ethics Statement

The study was approved by the Institutional Review Board at The University of Osaka Hospital (Approval No. 25243). The requirement for written informed consent was waived because the study only used anonymized patient information and was designed as a retrospective observational study with no interventions or invasive procedures. All procedures were performed following the ethical standards of the 1964 Helsinki Declaration and later versions.

## Conflicts of Interest

Hideki Endo and Hiroyuki Yamamoto are affiliated with the Department of Healthcare Quality Assessment of the University of Tokyo. The department is a social collaboration department supported by grants from the National Clinical Database, Intuitive Surgical Sarl, Johnson and Johnson K.K., and Nipro Co. The other authors declare no conflicts of interest.

## Data Availability

Data sharing not applicable to this article as no datasets were generated or analysed during the current study.
